# Reactivating latent HIV with PKC agonists induces resistance to apoptosis and is associated with phosphorylation and activation of BCL2

**DOI:** 10.1371/journal.ppat.1008906

**Published:** 2020-10-19

**Authors:** Andrea J. French, Sekar Natesampillai, Ashton Krogman, Cristina Correia, Kevin L. Peterson, Alecia Alto, Aswath P. Chandrasekar, Anisha Misra, Ying Li, Scott H. Kaufmann, Andrew D. Badley, Nathan W. Cummins

**Affiliations:** 1 Division of Infectious Diseases, Mayo Clinic, Rochester, Minnesota, United States of America; 2 Division of Oncology Research, Mayo Clinic, Rochester, Minnesota, United States of America; 3 Division of Biomedical Statistics and Informatics, Mayo Clinic, Rochester, Minnesota, United States of America; 4 Department of Molecular Pharmacology & Experimental Therapeutics, Mayo Clinic, Rochester, Minnesota, United States of America; 5 Department of Molecular Medicine, Mayo Clinic, Rochester, Minnesota, United States of America; Vaccine Research Center, UNITED STATES

## Abstract

Eradication of HIV-1 by the “kick and kill” strategy requires reactivation of latent virus to cause death of infected cells by either HIV-induced or immune-mediated apoptosis. To date this strategy has been unsuccessful, possibly due to insufficient cell death in reactivated cells to effectively reduce HIV-1 reservoir size. As a possible cause for this cell death resistance, we examined whether leading latency reversal agents (LRAs) affected apoptosis sensitivity of CD4 T cells. Multiple LRAs of different classes inhibited apoptosis in CD4 T cells. Protein kinase C (PKC) agonists bryostatin-1 and prostratin induced phosphorylation and enhanced neutralizing capability of the anti-apoptotic protein BCL2 in a PKC-dependent manner, leading to resistance to apoptosis induced by both intrinsic and extrinsic death stimuli. Furthermore, HIV-1 producing CD4 T cells expressed more BCL2 than uninfected cells, both *in vivo* and after *ex vivo* reactivation. Therefore, activation of BCL2 likely contributes to HIV-1 persistence after latency reversal with PKC agonists. The effects of LRAs on apoptosis sensitivity should be considered in designing HIV cure strategies predicated upon the “kick and kill” paradigm.

## Author summary

The major barrier to an HIV cure is the latent viral reservoir. We questioned why some drugs that reactivate latent HIV fail to reduce the viral reservoir size. We show that some HIV latency reversal agents, particularly PKC agonists such as bryostatin-1, activate the anti-apoptotic BCL2 protein in CD4 T cells. This unintended biologic effect inhibits apoptosis and, thereby, may promote HIV persistence despite viral reactivation. It is therefore important to screen potential latency reversal agents for off-target effects that might promote survival of HIV infected cells.

## Introduction

In 2017, nearly one million people died of AIDS-related illnesses worldwide. Despite the availability of effective anti-retroviral treatment (ART), lifelong ART is sometimes inaccessible, unaffordable, or not feasible due to adverse drug effects and drug-drug interactions (DDIs). An HIV-1 cure could eliminate the risks posed by lifelong ART and potentially end the spread of HIV-1 in populations with limited access to ART.

The “kick and kill” strategy to cure HIV-1 involves reactivating latent virus inside host cells, allowing either immune-mediated killing or HIV-induced death of the infected cells [1]. Currently, several latency reversal agents (LRAs) are being evaluated in clinical trials [2]. While some of these drugs (vorinostat, bryostatin-1, disulfiram, panobinostat, and romidepsin) did increase HIV-1 transcription, none significantly reduced the latent viral reservoir [3–7]. Thus, the induction of HIV-1 transcription alone is not sufficient to cause death of the reactivating cell.

While cell death in the context of untreated HIV-1 infection occurs in both infected and uninfected cells, an eradication cure would involve preferential death of HIV-infected cells, and could occur through the following non-exclusive pathways: pyroptosis [8], intrinsic and extrinsic apoptotic pathways [9], DNA-PK activation [10], and HIV protease-induced cleavage of procaspase 8 [11].

In HIV-1 infected cells, HIV-1 protease plays a significant role in the viral life cycle through its cleavage of the HIV-1 polymerase protein from Gag to create infectious virus and its ability to degrade the anti-apoptotic BCL2 protein, thereby tipping the balance in favor of cell death [12]. In addition, the HIV-1 protease can independently induce apoptosis of HIV protein transcribing, infected cells. In particular, HIV-1 protease has been shown to cleave procaspase 8 to a 41 kDa fragment termed Casp8p41 [13–15]. This enzymatically-inactive cleaved caspase can both bind and activate the mitochondrial permeabilizer BAK [16,17], or in case of high BCL2 levels, bind BCL2 instead and avert cell death [18]. Additionally, many proteins such as Nef and Tat act to upregulate FAS and FASL on the cell surface, thereby increasing sensitivity to stimuli that trigger the extrinsic apoptotic pathway [9]. CD8 T cells recognize the FAS on infected CD4 T cells and kill these infected cells. In cases where HIV-1 infection has caused downregulation of MHC I, NK cells can detect this absence and kill infected cells [19,20].

To reactivate HIV from latency, investigational LRAs target a wide array of signaling pathways, including histone deacetylases (vorinostat, panobinostat, valproic acid and romidepsin), protein kinase C isoforms (bryostatin-1 and prostratin), proteasome mediated protein degradation (ixazomib), CyclinT1/p-TEFb (JQ1), and PTEN/PI3K (disulfiram). Because of the many cellular processes affected by these pathways, these LRAs have the potential for unintended effects other than stimulation of HIV transcription [4,21–23]. PKC agonists such as bryostatin-1 activate IκB kinase to induce phosphorylation of IκB, which is degraded to allow enhanced NFκB signaling [21]. This regulation of NFκB impacts viral transcription through the NFκB promoters in the enhancer region of HIV-1 long terminal repeats [24]. NFκB also regulates cell survival both in the intrinsic and extrinsic cell death pathways through transcriptional regulation of key apoptosis proteins, including the anti-apoptotic BCL2 family members BCL2 and BCLX_L_, among others [25].

Given the pleiotropic transcriptional activities induced by LRAs and the numerous signaling pathways they impact, we sought to understand how different LRAs impact cell death pathways and determine whether the choice of LRA impacts the ability of HIV-infected cells to live or die following reactivation.

## Results

### Some latency reversal agents inhibit apoptosis

**Fig 1:**
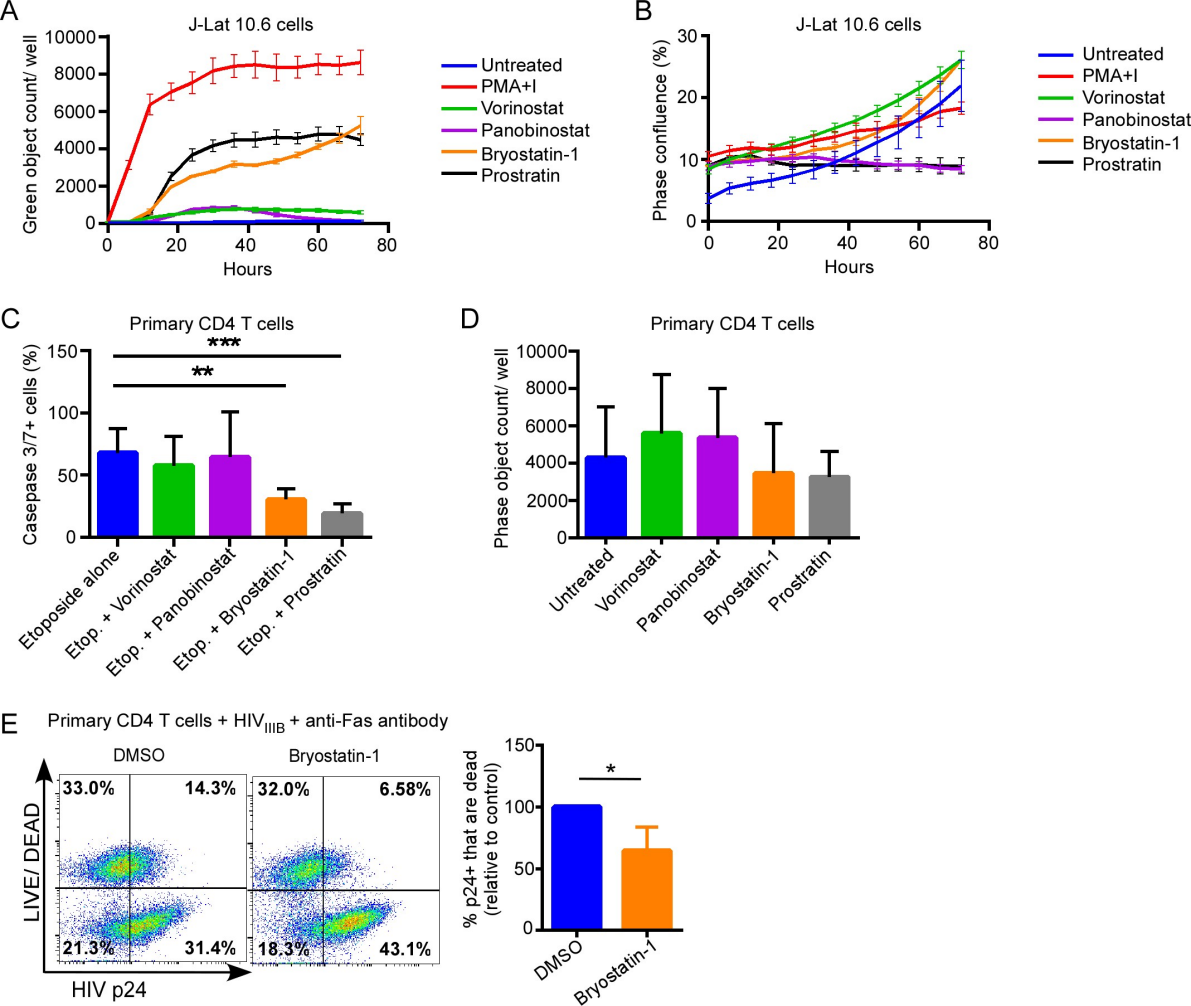
PKC agonists reduce apoptosis sensitivity in CD4 T cells. A) Chronically HIV infected J-Lat 10.6 cells were treated with the LRAs indicated and expression of GFP monitored over time using live cell imaging. Data represent mean (error) of 4 assay replicates of an representative experiment from 3 independent experiments. B) J-Lat 10.6 cells treated as in (A) were monitored for proliferation by phase confluence using live cell imaging. C) Uninfected primary CD4 T cells were treated with LRAs or diluent control for 24 hours, supplemented with etoposide (20 μM), and assessed for apoptosis at 72 hours by measurement of caspase 3/7 activity. Data represent mean (SD) of 4 assay replicates from 3 independent experiments. D) Uninfected primary CD4 T cells, as in (C), were treated with LRAs and assessed for cell proliferation by absolute cell counts at 72 hours. E) Activated primary CD4 T cells were infected with HIV_IIIB_ for 48 hours, treated with DMSO or bryostatin-1 (10 ng/ml) followed by anti-Fas antibody treatment (Clone CH11, 0.5–1.0 μg/ml). LIVE/DEAD viability stain and HIV p24 expression were measured by flow cytometry. Representative results as well as mean (SD) of 3 independent experiments are shown. *** p<0.0001; ** p<0.001; * p<0.05.

We questioned whether LRAs might inhibit apoptosis induced by a variety of different stimuli. First, we treated J-Lat 10.6 cells, a Jurkat T cell based line containing an integrated HIV-1 genome that expresses green fluorescent protein (GFP) on activation [26], with a panel of LRAs and monitored GFP expression over time (Fig 1A). Untreated J-Lat 10.6 cells expressed GFP rarely, whereas PMA+Ionomycin induced rapid and robust GFP expression in treated J-Lat 10.6 cells, consistent with maximal HIV latency reversal [27]. Bryostatin-1 and prostratin induced moderate GFP expression, whereas vorinostat and panobinostat induced GFP expression modestly, consistent with previous reports [27]. None of the LRAs tested significantly increased proliferation of the J-Lat 10.6 cells (Fig 1B), confirming that the increase in number of GFP expressing cells was due to reactivation of latent HIV and not enhanced proliferation.

Having confirmed the latency reversal activity of these LRAs, we then examined the effects of the LRAs on apoptosis sensitivity, first in uninfected primary CD4 T cells. Primary CD4 T cells from 3 healthy donors were pre-treated with LRAs at physiologic doses for 24 hours followed by treatment with etoposide, which causes DNA damage and results in cell death by the intrinsic apoptotic pathway [28,29]. Activation of caspases-3 and -7, final common effectors of apoptotic pathways [30,31], was monitored by live cell imaging at 72hrs in treated cells (Fig 1C). Pretreatment of CD4 T cells with bryostatin-1 (p = 0.0004) or prostratin (p<0.0001) blunted etoposide-induced killing, whereas the HDAC inhibitors vorinostat and panobinostat did not significantly alter etoposide-induced caspase-3/7 activity (Fig 1C), without inducing significant proliferation in similarly treated cells (Fig 1D). This suggests that some, but not all, LRAs inhibit caspase-3/7 activation in CD4 T cells through the intrinsic apoptosis cascade.

Because HIV-1 infection also sensitizes CD4 T cells to extrinsic apoptosis induced by FAS ligation [32,33], we also questioned whether bryostatin-1 would inhibit FAS-mediated apoptosis in HIV-infected cells. Pretreatment of acutely HIV_IIIB_ infected primary CD4 T cells with bryostatin-1 resulted in significantly reduced cell death of HIV-p24 positive cells after FAS ligation compared to vehicle control treated cells (35.6 ± 11.2% relative reduction, p = 0.034, Fig 1E). Therefore, bryostatin-1 also has a protective effect against extrinsic apoptosis in HIV-infected cells.

In summary, our data indicate that some of the current investigational LRAs modulate susceptibility of primary CD4 T cells to apoptosis inducing treatments, including in HIV infection. Specifically, PKC agonists protect against apoptosis induced through both the intrinsic and extrinsic apoptotic pathways, which have been implicated in HIV-induced cell death.

### PKC agonists activate anti-apoptotic BCL2 through ERK-dependent phosphorylation

**Fig 2:**
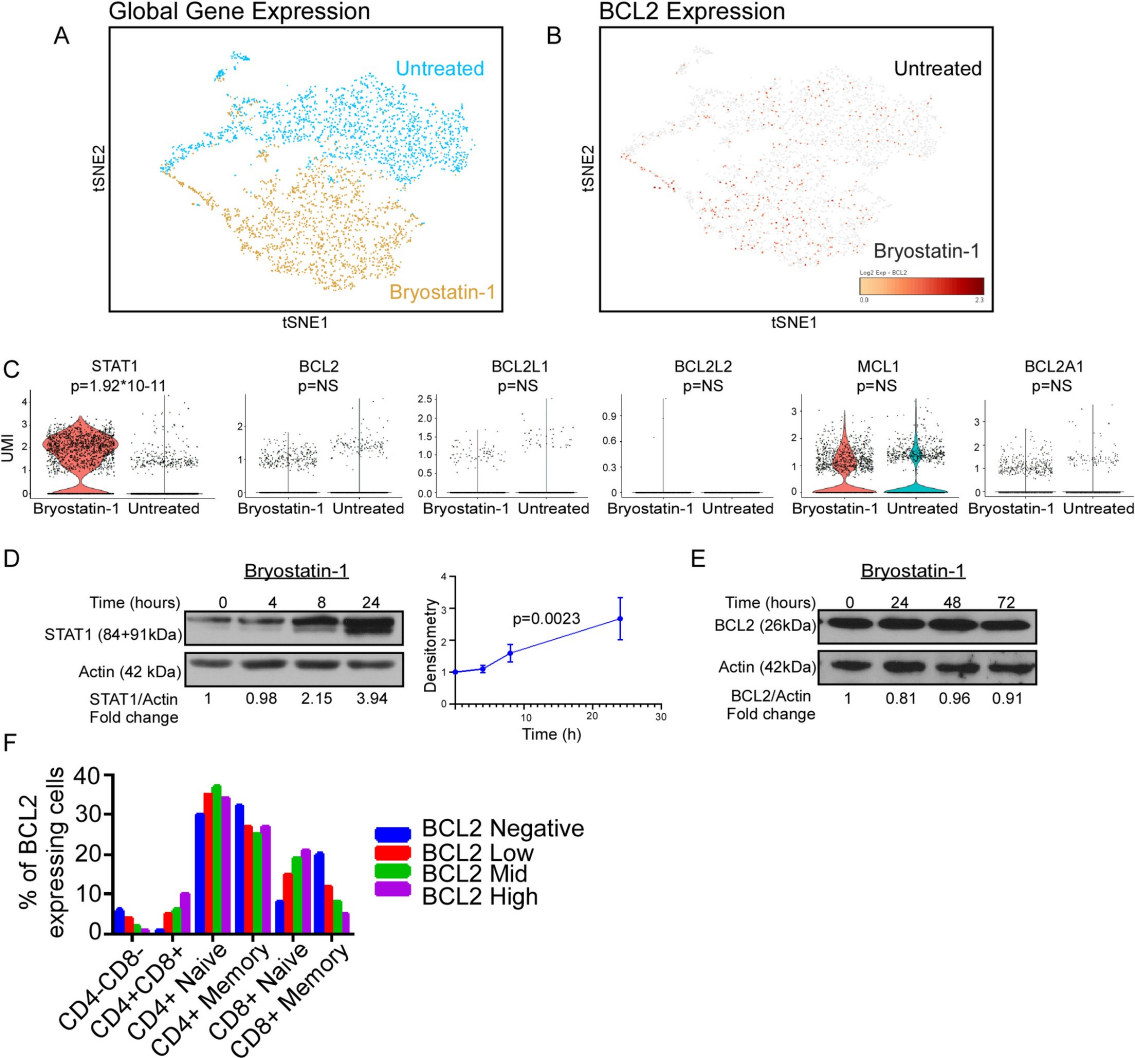
Cell-to-cell variability in BCL2 expression in CD4 T cells. A-C) Single-cell RNAseq (scRNA) was performed on primary CD4 T cells from an ART-suppressed, HIV positive donor treated with bryostatin or control for 24 hours. A) tSNE plot of whole transcriptome scRNA sequencing showing global gene expression differences. B) tSNE plot showing BCL2 positive cells (brown) from both the untreated and bryostatin-treated CD4 T cells. C) Violin plots comparing expression of STAT1 and anti-apoptotic BCL2 family proteins between cells treated with bryostatin and untreated cells. D-E) Western blots showing changes: D) in STAT1 levels over 24 hours in CD4 T cells with graph showing changes of STAT1 over time (STAT1/actin ratio across 3 replicates). Significance (p<0.0023) was determined using a linear regression with the slope being significantly non-zero. E) BCL2 expression of bryostatin-treated CD4 T cells over 72 hours. F) Flow cytometry for intracellular BCL2 expression in T cell subsets was performed. Depicted is aggregated data from (n = 3) HIV positive subjects.

To search for the mechanism(s) by which PKC agonists inhibit apoptotic signaling, we performed single cell RNAseq (scRNAseq Chromium 10X). Here, we focused on apoptotic-regulatory gene expression alterations in primary CD4 T cells isolated from an ART-suppressed, HIV-positive patient treated with the PKC agonist bryostatin-1 vs. control (Fig 2A). Of the entire data set, eight genes had a unique molecular identifier (UMI) count greater than one transcript per cell and were significantly differentially expressed (log2 fold change > 1) in bryostatin-1 treated cells compared to control (Table 1). Gene ontology analysis of the differentially expressed genes (PANTHER Overrepresentation Test from the GO Ontology database, released 2019-02-02) revealed enrichment in the following biological processes: positive regulation of cytokine production (p = 1.52E-05), regulation of immune response (p = 2.33E-05), type I interferon signaling pathway (p = 1.96E-06), interferon-gamma-mediated signaling pathway (p = 1.10E-08), and the cellular response to interferon-beta (p = 3.20E-05). Surprisingly, no significant changes in anti-apoptotic BCL2-family gene expression [BCL2, BCL2L1 (BCLXL), BCL2L2 (BCLW), MCL1, or BCL2A1] were detected (Fig 2B and 2C).

**Table 1:** Differentially expressed genes in primary CD4 T cells treated with bryostatin or diluent.

GeneName	Bryostatin Average UMI	Untreated Average UMI	Log2 Fold Change	P-Value
GBP5	1.06882563	0.062958256	4.070992895	4.78E-14
STAT1	2.370611622	0.212568736	3.475283279	1.92E-11
MT2A	3.514036918	0.448154469	2.969409885	9.52E-09
GBP2	3.104829391	0.628228622	2.304148409	1.27E-05
CD74	1.969256691	0.532098811	1.886717424	0.001646
ACTG1	6.883673312	2.006540554	1.778420173	0.001679
ID3	1.053556692	0.328330691	1.680019213	0.004494
IRF1	1.106779846	0.434615059	1.347228404	0.043816

UMI: unique molecular identifier

Results from the scRNAseq experiment were validated in three independent ways. First, one of the most differentially expressed genes in response to bryostatin treatment was STAT1 (log2 fold change > 3, p = 1.92*10^−11^, Fig 2C). In a parallel experiment, we monitored STAT1 protein expression in primary CD4 T cells treated with bryostatin-1 vs. diluent to show that STAT1 protein expression significantly increased over time (Fig 2D). Second, primary CD4 T cells treated with bryostatin-1 did not express more BCL2 protein over time as monitored by western blot (Fig 2E), consistent with our scRNAseq data. Third, PBMCs from HIV-1 positive donors were assessed for intracellular BCL2 expression in T cell subsets (Fig 2F). Consistent with the scRNA gene expression data, not every T cell expressed BCL2 protein. Notably, there were significant differences in frequency of naïve and memory CD4 and CD8 T cell subsets within BCL2 expressing and non-expressing cells (p = 0.0080).

**Fig 3:**
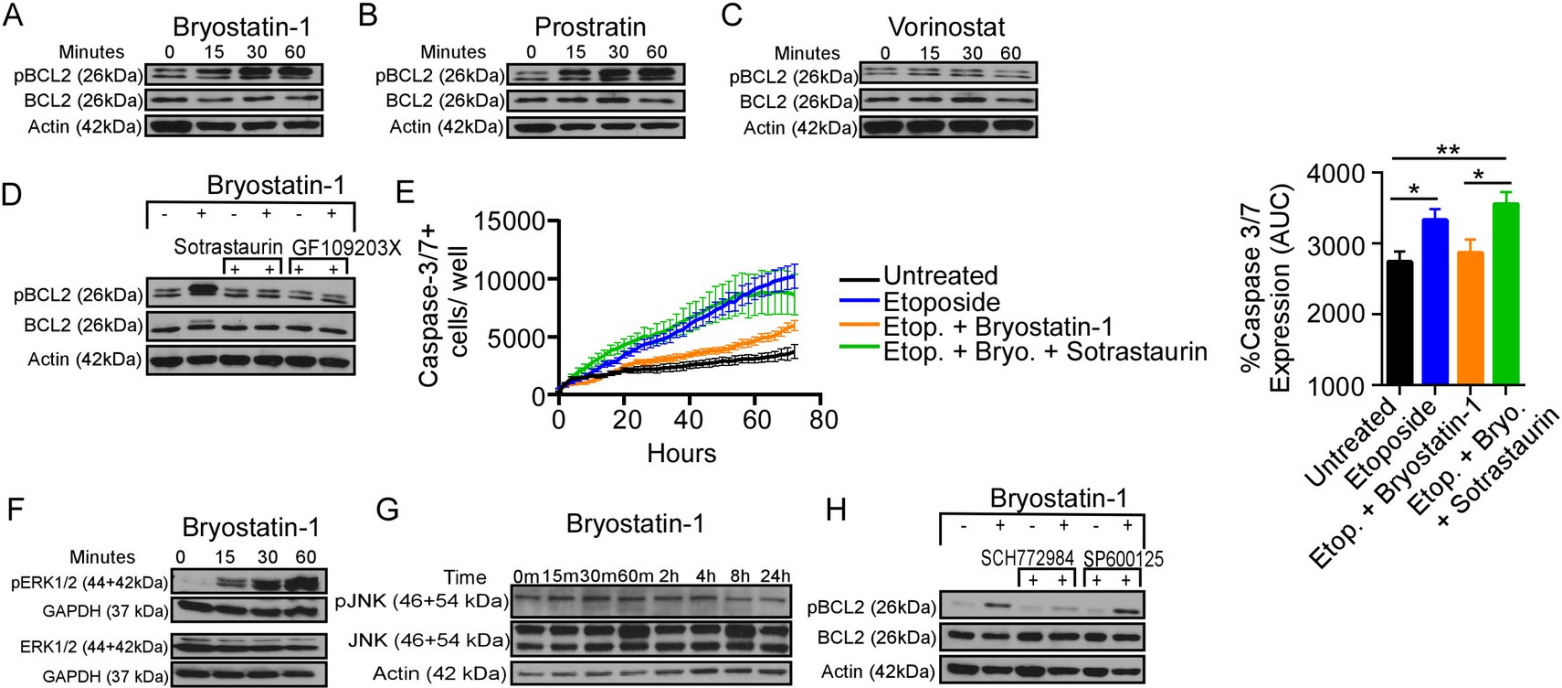
PKC agonists induce ERK-dependent BCL2 phosphorylation. A-C) CD4 T cells treated with 3 different LRAs (A: bryostatin-1; B: prostratin; C: vorinostat) were assayed for phospho-S70-BCL2 and total BCL2 by western blot. D) Cells treated with PKC inhibitors GF109203X (GF) or Sotrastaurin prior to treatment with bryostatin-1 were assessed for phosphorylation of BCL2 S70. E) CD4 T cells were treated with the PKC inhibitor sotrastaurin prior to treatment with bryostatin-1 and etoposide (ETP). Cells were tracked over 3 days for active caspase 3/7. Graphs show mean (SD) area under the curve for each treatment. * p<0.05; ** p<0.001. F-G) CD4 T cells treated with bryostatin-1 over an hour or over 24 hours were probed for phospho-ERK1/2 (F) or phospho-JNK (G). Western blots showed increased ERK1/2 phosphorylation but not JNK phosphorylation. H) CD4 T cells treated with ERK1/2 inhibitor SCH772984 or JNK inhibitor SP600125 (SP) and bryostatin-1 showed loss of BCL2 phosphorylation with SCH772984 but not SP600125.

Protein function can also be modified independent of changes in transcription. Previous studies have shown that BCL2 can be phosphorylated at S70, which increases the ability of BCL2 to inhibit apoptosis through increased binding to the pro-apoptotic proteins [34,35]. Bryostatin-1 induces phosphorylation of BCL2 at S70 in murine cells [36] and in human leukemic pre-B cells [37]. However, it remains unknown whether PKC agonists inhibit apoptosis by inducing BCL2 S70 phosphorylation in human CD4 T cells. To investigate this possibility, we first used primary CD4 T cells from three different donors that were treated with bryostatin or prostratin or the HDAC inhibitor vorinostat (which did not inhibit apoptosis) and monitored BCL2-S70 phosphorylation by western blot. Both PKC agonists bryostatin and prostratin significantly increased phosphorylation of BCL2 over time (Fig 3A and 3B, p = 0.0009 and p = 0.02, respectively), whereas vorinostat did not (Fig 3C). Taken together, these data indicate that PKC activators, but not all types of LRAs, induce phosphorylation of BCL2 at S70 in human CD4 T cells (Fig 3A–3C).

PKC can signal through ERK or JNK [35], and ERK1/2 is able to directly phosphorylate BCL2 at S70 [38]. To determine which pathway bryostatin-1 and prostratin use to phosphorylate BCL2 in CD4 T cells, we pre-treated CD4 T cells with two different PKC inhibitors, sotrastaurin or GF109203X [39,40]; with an ERK1/2 inhibitor, SCH772984; or with a broad-spectrum JNK inhibitor, SP600125 [41,42], for one hour prior to treating with bryostatin and assessing BCL2 phosphorylation status. As expected, both PKC inhibitors prevented bryostatin-1-induced BCL2 phosphorylation (Fig 3D), indicating that PKC activity is required for bryostatin-1-induced BCL2 phosphorylation (sotrastaurin p = 0.02, and GF109203X p = 0.04).

To confirm that bryostatin-1 was inducing apoptosis resistance through PKC activation, cells were pre-treated with the PKC inhibitor sotrastaurin for one hour, bryostatin-1 and etoposide were added, and cells were imaged for active caspases 3 and 7 over 72 hours. The sotrastaurin reversed the resistance to apoptosis induced by bryostatin-1, as cells that were treated with bryostatin-1 and the PKC inhibitor activated similar amounts of caspases 3/7 over time as cells that were only treated with etoposide (Fig 3E).

CD4 T cells treated with bryostatin-1 showed rapid ERK1/2 phosphorylation (Fig 3F), but no JNK phosphorylation within 24 hours (Fig 3G). Furthermore, treatment with the ERK1/2 inhibitor decreased bryostatin-1-induced BCL2 phosphorylation, whereas the JNK inhibitor did not (Fig 3H, p = 0.008 and n.s., respectively). Together, these data suggest that PKC agonist-induced BCL2 phosphorylation at S70 is dependent upon ERK1/2, but not JNK, and occurs downstream of PKC activation.

### Phospho-BCL2-S70 has increased affinity for Casp8p41

**Fig 4:**
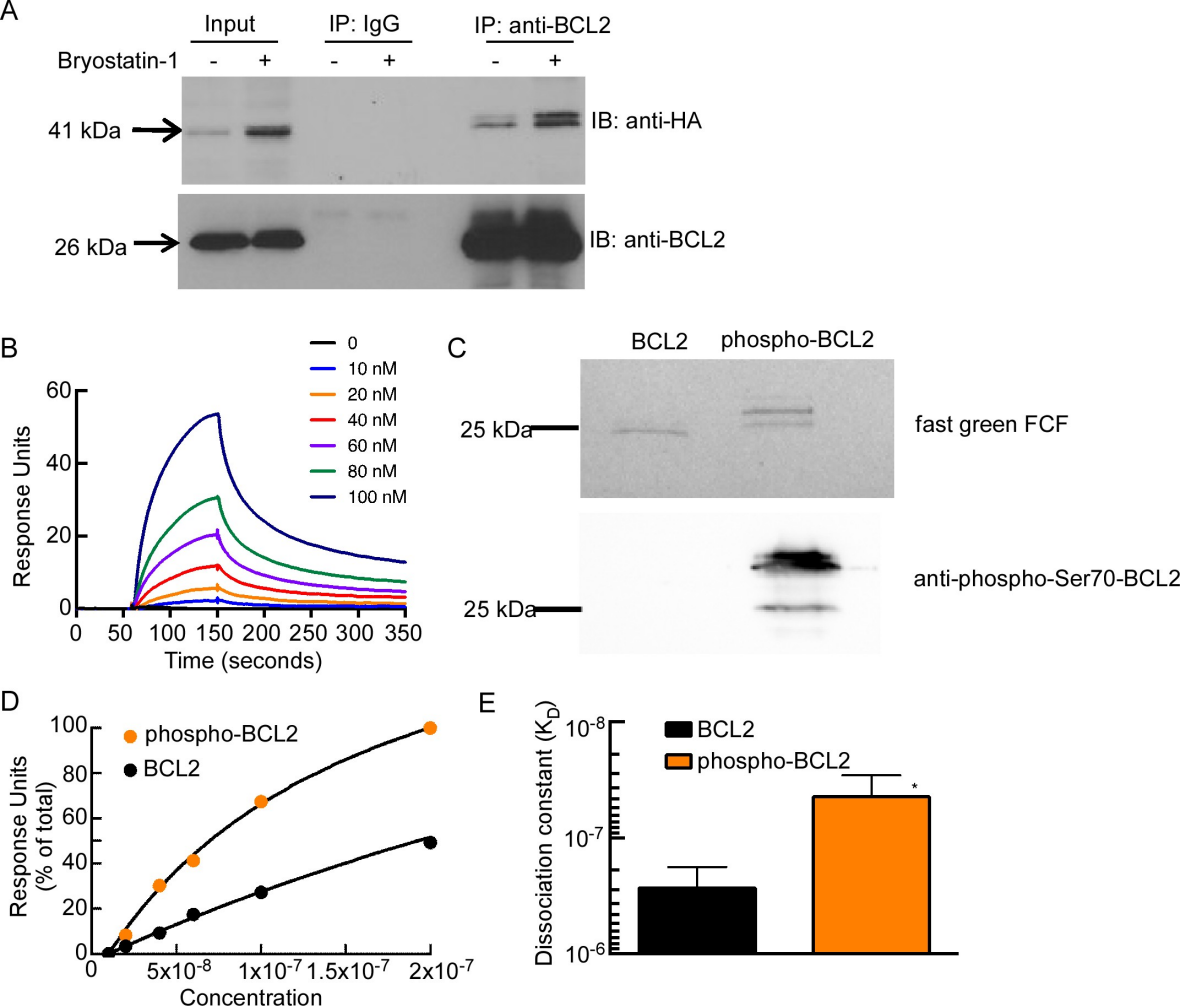
BCL2 phosphorylation enhances interaction with Casp8p41. (A) Primary CD4 T cells were transfected with HA-Casp8p41 followed by treatment with bryostatin or vehicle control. Immunoprecipitation (IP) was performed for BCL2 or control, and immunoblot (IB) for HA and BCL2. (B) SPR sensorgrams showing the dose-dependent binding of BCL2 (0–100 nM) to Casp8p41. (C) Purified BCL2ΔTM-His6 was phosphorylated by CDK1/cyclin B *in vitro* (see Materials and Methods). The mixture was separated by SDS-PAGE, transferred to nitrocellulose, and stained with fast green FCF, or blotted with anti-phospho-Ser70-BCL2 antibody. (D) Affinity curves showing the dose-dependent binding of BCL2 and phospho-BCL2-S70 (0–100 nM) to Casp8p41, as indicated by percentage of total response units (RUs) after background subtraction. (E) Affinities of BCL2 and phospho-BCL2-S70 for Casp8p41 were calculated. P < 0.0106 versus control by unpaired t-test. Error bars, SD of three independent experiments.

Previous studies have shown that phosphorylation on S70 increases the ability of BCL2 to bind pro-apoptotic BCL2 family members in intact cells and under cell-free conditions. We have previously reported that HIV protease, when expressed in HIV-infected cells [14], including those reactivated from latency [18], cleaves cellular procaspase 8 to generate Casp8p41, an enzymatically inactive N-terminal fragment that induces apoptosis by binding and activating BAK at the mitochondrial outer membrane [16,17]. Because Casp8p41 can be bound and inactivated by BCL2 [18], we asked whether BCL2 S70 phosphorylation also impacts the interaction of BCL2 with Casp8p41 in cells. Primary CD4 T cells were transfected with HA-Casp8p41 in the presence or absence of bryostatin-1, and immunoprecipitation for BCL2 was performed. Indeed, bryostatin-1 increased the amount of HA-Casp8p41 bound to BCL2 compared to control treatment (Fig 4A).

We next sought to determine if phosphorylation of BCL2 strengthened the interaction of BCL2 with Casp8p41. To do this, we used surface plasmon resonance spectroscopy [18] to compare the interaction of Casp8p41 with unphosphorylated and S70-phosphorylated BCL2. As we have previously demonstrated [18], BCL2 binds to immobilized Casp8p41 (Fig 4B). Next, BCL2 was phosphorylated *in vitro*, as confirmed by immunoblotting with antibody to phosphor-S70-BCL2 (Fig 4C). Phosphorylated BCL2 interacted more tightly (6.5-fold decrease in K_D_ from 260 ± 8 nM to 40 ± 2 nM) with Casp8p41 (Fig 4D and 4E), highlighting the potential importance of this post-translation modification in modulating the neutralization of Casp8p41 and other pro-apoptotic proteins during HIV reactivation in CD4 T cells.

### HIV infected cells have higher BCL2 than uninfected cells

**Fig 5:**
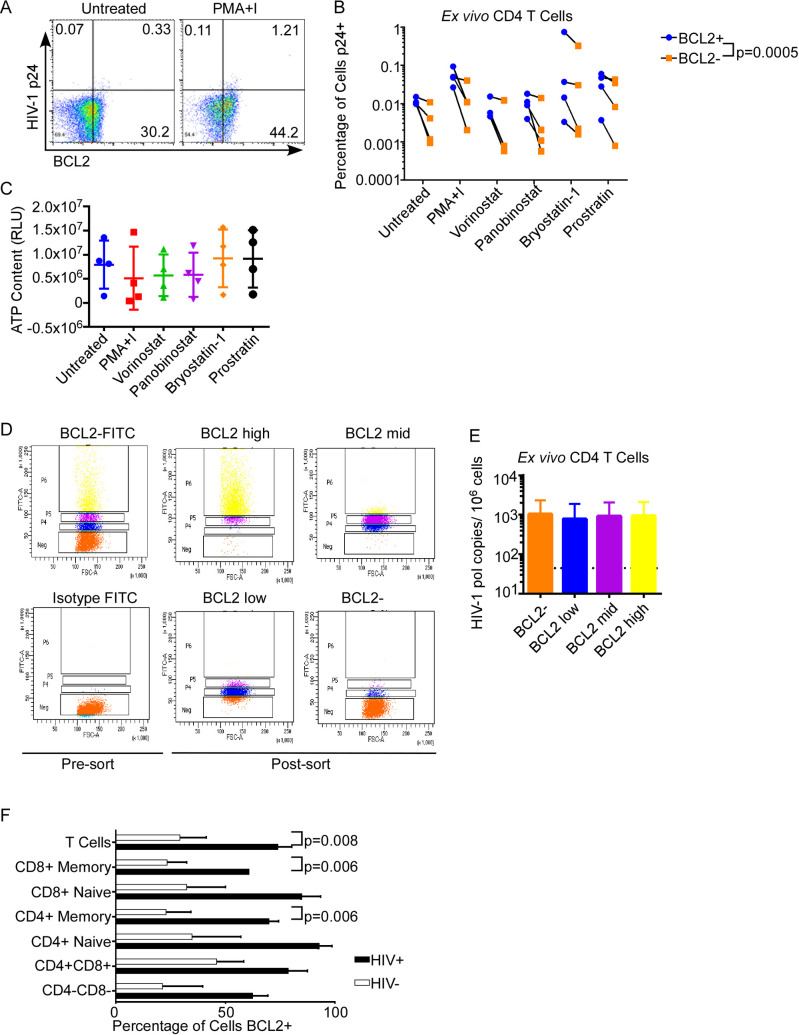
BCL2+ T cells Occur More in HIV-1^+^ Donors than in Uninfected Donors and Express More p24 than BCL2- cells. A-B) Primary CD4 T cells from HIV positive donors were treated *ex vivo* with a panel of LRAs at physiologic concentrations, and assessed for intracellular BCL2 and HIV-p24 expression by flow cytometry. Representative FACS plots on unstimulated and maximally stimulated cells are in (A). Full data are represented in (B). A ratio paired t test comparing BCL2+ cells and BCL2- cells in all treatment conditions was used to assess statistical significance. C) Potential LRA-induced proliferation or toxicity was assessed in the treated CD4 T cells from (A-B) by measuring ATP content. D-E) *Ex vivo* CD4 T cells from 4 ART-suppressed, HIV positive donors were FACS sorted by intracellular BCL2 expression (D) and total HIV DNA measured in sorted cells by digital droplet PCR (E). F) PBMCs from healthy donors and HIV-positive persons were stained for T cell subsets using Live/Dead, CD3, CD4, CD8, CD27, and CD45RO. BCL2 expression in T cell subsets from healthy donors and HIV-infected persons were compared. Statistical significance was determined using multiple t tests.

Our previous data indicate that PKC agonists such as bryostatin can inhibit apoptosis in CD4 T cells through BCL2 S70 phosphorylation and activation. However, we have also demonstrated that not all CD4 T cells express BCL2 (Fig 2B, 2C and 2F). Since PKC agonists do not increase BCL2 expression in CD4 T cells, BCL2 activation by PKC agonists would only have relevance to HIV persistence if HIV-infected and/or producing cells express BCL2 independent of LRAs. To investigate the latter possibility, primary CD4 T cells from ART-suppressed HIV-positive subjects were treated with LRAs for 24 hours then co-stained for intracellular HIV p24 (a marker of HIV transcription and translation) and BCL2. HIV p24 staining occurred more frequently in BCL2+ compared to BCL2- CD4 T cells (p = 0.0005, Fig 5A and 5B). Importantly, treatment with LRAs did not induce non-specific proliferation or toxicity in treated cells at 24 hrs (Fig 5C). This suggests that HIV is expressed preferentially but not exclusively in BCL2+ cells.

Next, we questioned whether HIV infection was present more in BCL2+ cells compared to BCL2- cells. To assess this, we sorted primary CD4 T cells from ART-suppressed HIV-positive subjects (a setting of minimal HIV replication) based on intracellular BCL2 expression (Fig 5D) and measured total HIV-1 DNA in sorted cells by digital droplet PCR (Fig 5E). There was no difference in HIV-1 DNA content between cells with no or low BCL2 expression or high BCL2 expression. Together, these data suggest that viral production (monitored by p24 expression) occurs more frequently in BCL2+ cells than BCL2- cells despite a similar frequency of infection (HIV DNA).

Even under suppressive ART, HIV infection causes aberrant immune activation that is associated with increased NFκB signaling. Because NFκB can drive BCL2 expression as noted above, we asked whether T cells from HIV positive patients might express more BCL2 than healthy controls. In fact, the proportion of T cells with high BCL2 was significantly greater in CD8^+^ Memory T cells (p = 0.003), CD4^+^ memory T cells (p = 0.002), and total T cells (p = 0.004), from HIV positive subjects compared to controls (Fig 5F).

## Discussion

Results of the present study provide a number of observations that help inform current efforts to selectively kill HIV-infected cells by viral reactivation. First, CD4 T cells express variable amounts of the anti-apoptotic BCL2 protein, and CD4 T cells from HIV positive patients express high levels of BCL2 more frequently than from HIV negative patients. Second, HIV production is, on the whole, higher in BCL2+ CD4 T cells than in BCL2- T cells despite a similar infection frequency, suggesting but not proving the possibility that BCL2 expression favors survival of reactivated, virus producing cells. Third, some latency reversal agents induce resistance to apoptosis; in the case of PKC agonists, this is associated with ERK-dependent BCL2 phosphorylation. Taken together, these observations raise concern that a “kick and kill” strategy, if it is to succeed, must take into account the intrinsic apoptotic milieu of the infected cell and the potential that an unintended consequence of some LRAs is protection of reactivated or reactivating infected cells from cell death.

Thus far, the kick and kill strategy of HIV cure has focused on identifying or creating a therapeutic compound that can effectively reactivate HIV-1 from latently infected cells, then allowing the reactivating cell to be killed by host immune mechanisms or through the intrinsic pro-apoptotic effects of HIV replication. A number of agents are showing promise as latency reactivators. However, these agents are not sufficiently active on their own to markedly diminish the HIV reservoir, prompting speculation that LRAs will need to be used in combination with either immune boosting interventions (i.e. therapeutic vaccination, broadly neutralizing antibodies, or immune activators such as IL-15), or compounds that promote the death of HIV infected cells which reactivate (e.g., ixazomib or venetoclax) [18,22,43].

In addition to off-target effects, i.e., unanticipated biologic activities that result from inhibition of a target other than the intended target and are contrary to the desired therapeutic purpose, new agents or even repurposed drugs can also have unwanted on-target effects. The induction of tumor lysis syndrome by venetoclax in chronic lymphocytic leukemia, for example, represents on-target but unwanted effects [44]. Our current report identifies an unwanted on-target biologic effect of PKC agonists that run contrary to the desired intent of inducing all HIV infected cells to die. Previous studies have focused on the individual toxicities of LRAs on effector cells [23], and not how the LRAs might impact induced cell death in the reactivated cell. Inhibition of apoptosis by PKC agonists suggests that they too might alter cell death induced by reactivated HIV, or cell death induced by activated NK or CD8 T cells against an otherwise susceptible target cell. These unintended effects could contribute to the observed failure of the “kick and kill” strategy to date. As novel LRAs continue to be described, it will be critical to more thoroughly identify the biological activities of these drugs prior to initiating clinical trials.

Efforts to determine the cause of the anti-apoptotic effects of PKC agonists through RNAseq were unsuccessful. Instead, the answer came from considering posttranslational modifications in BCL2 family members. While PKC-activated pathways have been previously shown to impact degradation rates or activity of the BCL2 family proteins MCL1 [45,46] and BCLX_L_ [47,48], the present study showed that PKC-family proteins impact BCL2 as well. In particular, treatment with bryostatin-1 or prostratin enhances BCL2 S70 phosphorylation, a modification that has been previously documented to increase the anti-apoptotic activity of BCL2 after cytokine treatment [35,36]. This BCL2 S70 phosphorylation results in a 6-fold increase affinity of BCL2 for Casp8p41, a pro-apoptotic protein generated by HIV-1 protease in cells with virus. This could help explain why bryostatin-1 promoted one of the highest levels of viral reactivation in the JLAT 10.6 model for latently-infected cells (Fig 1A) but was not successful at reducing viral loads *in vivo* [5]. Unfortunately, vorinostat, which does not inhibit CD4 T cell apoptosis or activate BCL2, is not a sufficiently potent LRA to reduce HIV reservoir size on its own [3,49] *in vivo*.

The impact of bryostatin-1-induced BCL2 activation might be especially potent in HIV positive persons. It has previously been shown that individual HIV proteins (such as Tat) can promote an anti-apoptotic state through increased BCL2 expression [50] as well as increased NF-κB signaling [51]. Consistent with these observations, our results and the work of multiple others [52–55] indicate that BCL2 expression in CD4 T cells is higher in ART-suppressed HIV positive persons. Our data suggest that bryostatin-1 may increase the survival of Casp8p41-expressing cells by increasing the ability of BCL2 to bind [18–22] and neutralize Casp8p41 (Fig 4). Because BCL2 inhibits multiple apoptotic pathways in the context of HIV infection [56], it is likely that pathways other than Casp8p41-mediated intrinsic apoptosis are also inhibited by bryostatin-1. Consistent with this notion, we observed that bryostatin-1 also inhibits FAS-induced apoptosis (Fig 1E).

There are several potential limitations of our study. First, PKC has different isoforms, and it is unclear which isoform is responsible for the anti-apoptotic effect of bryostatin-1 in our study [57]. Second, it is possible that bryostatin-1’s effect on apoptosis may be concentration, cell type and context dependent [58]. Finally, other bryostatin analogues may have varying effects on apoptosis sensitivity. All of these questions are worthy of future investigations.

In conclusion, the current study suggests another important caveat to the “kick and kill” strategy for HIV elimination, specifically the potential for certain LRAs to induce biochemical changes that enhance survival of reactivated cells. These potential adverse effects should be systematically evaluated in all novel potential latency reversal agents in order to ensure that the “kill” of HIV infected cells remains a possible goal.

## Materials and methods

### Cell isolation and culture

Primary uninfected PBMCs were isolated from leukocyte reduction system chambers [59] and CD4 T cells were isolated using the RosetteSep CD4 negative selection kit (Stem Cell Technologies, Vancouver, Canada) per the manufacturer’s protocol. Cells were then separated using Ficoll Paque density gradient centrifugation. Cells were cultured in RPMI 1640 medium with 10% FBS, 2 mM L-glutamine, 100 units/mL penicillin and 100 μg/mL streptomycin (complete RPMI). PBMCs from HIV positive subjects were obtained through two Mayo Clinic-approved IRB protocols (13–005646 and 16–001938). All subjects were virologically suppressed on combination antiretroviral therapy and provided written informed consent. PBMCs were isolated from leukapheresis samples using a Ficoll Paque density gradient. CD4s were isolated from PBMCs using an EasySep CD4 enrichment negative selection kit or RosetteSep CD4 enrichment kit (Stem Cell Technologies) and by following the manufacturer’s protocol. J-Lat 10.6 cells [26] were obtained from the NIH AIDS Reagent Program (Catalogue #9849).

### Key reagents

For all experiments, latency reversal agents (LRAs) were used at the following concentrations: 50 ng/mL PMA, 500 ng/mL Ionomycin, 500 nM vorinostat, 15 nM panobinostat, 10 ng/mL bryostatin-1, 1 μM prostratin, based on previous literature [60–63]. Cellular ATP measurement was performed using the CellTiter-Glo Luminescent Cell Viability Assay (Promega) per manufacturer’s protocol.

### IncuCyte live-cell imaging

96-well plates were coated using poly-L-ornithine and washed with sterile PBS. Cell cultures were diluted to 5 x 10^4^ or 2 x 10^5^ cells/mL using complete RPMI and pre-treated for 24 hours with LRAs. In experiments involving PKC inhibitors, cells were cultured overnight in RPMI 1640 medium with 2% human AB serum, 2 mM L-glutamine, and 100 units/mL penicillin (RPMI-AB). Prior to plating cell death experiments, IncuCyte Caspase 3/7 reagent was added to culture at a 1:3000 dilution. 200 μL of cell culture were plated per well in each well. Imaging was done every 2 hours for the duration of the experiment, up to 72 hours. Images were analyzed using IncuCyte Zoom software (Essen Bioscience Inc., GUI version 2018A).

### HIV infection experiments

CD4 T-Cells were isolated from a donor sample using the Rosette Sep negative selection kit (Stem Cell Technologies, Vancouver, Canada). Autologous NK cells were isolated from the same blood sample using the Rosette Sep Negative selection kit (Stem Cell Technologies, Vancouver, Canada). CD4 T-cells were activated for 48 hours in complete RPMI supplemented with 2 μg/ml PHA and 50 U/ml IL-2. The cells were washed and subsequently infected with HIV IIIB (NIH AIDS reagent) for 48 hours. NK Cells were maintained in RPMI + 50U/ml IL-2 at 37° C and the medium was refreshed at 72 hours. At 96 hours, the CD4 T-Cells were washed and re-suspended in fresh medium at a concentration of 1x10^6^ cells/ml. The cells were cultured for six hours with either no treatment, DMSO (vehicle control) or 10 ng/ml bryostatin. Subsequently, 1x10^6^ cells were cultured per condition either alone or with anti-Fas agonistic CH11 antibody at a dose of 1 μg/ml or 500 ng/ml. The dose of 10 ng/ml bryostatin was maintained throughout the duration of the experiment. The cells were collected at 48 hours post co-culture and stained with Live/Dead FITC fixable stain (Invitrogen) and fixed overnight with 2% PFA. The cells were subsequently permeabilized with 0.1% NP40, stained intracellularly with p24-PE mouse monoclonal antibody (Clone KC57, Beckman Coulter) and fixed with 2% PFA and analyzed by flow cytometry.

Digital droplet PCR (ddPCR) for HIV DNA was measured as previously described [22].

### Protein expression and purification

Plasmids encoding glutathione S-transferase (GST)-Casp8p41 in pGEX and His_6_-tagged-tagged Bcl-2ΔTM in pET29b have been described previously [15,64]. To express tagged Casp8p41 [17] or Bcl-2ΔTM, plasmids were transformed into *E*. *coli* BL21 by heat shock. After cells were grown to an optical density of 0.8, 1 mmol/L IPTG (isopropyl-l-thio-β-1-D-galactopyranoside) was added to the induce protein synthesis at 18°C for 24 hours. Bacteria were frozen and thawed on ice; suspended in calcium- and magnesium-free Dulbecco’s phosphate buffered saline (PBS) containing 0.1% Triton X-100, and 1 mM PMSF (GST-tagged proteins). Alternatively, bacteria expressing His_6_-tagged proteins were then washed and sonicated on ice in TS buffer [150 mmol/L NaCl containing 10 mmol/L Tris-HCl (pH 7.4) and 1 mmol/L freshly added PMSF]. All further steps were performed at 4°C. After His_6_-tagged proteins were applied to Ni^2+^-NTA-agarose, columns were washed with 20 volumes of TS buffer followed by 10 volumes of TS buffer containing 40 mmol/L imidazole and eluted with TS buffer containing 200 mmol/L imidazole. After GST-tagged proteins were incubated with GSH-agarose overnight, beads were washed twice with 20–25 volumes of TS buffer and eluted twice with TS containing 20 mmol/L GSH for 30 minutes at 4°C.

### *In vitro* phosphorylation and mass spectrometry

To achieve a high degree of modification at Ser^70^, 2.5 μg purified BCL2ΔTM-His6 was incubated with 1 μg purified CDK1/cyclin B complex (Millipore, cat 14–450) at 30°C for 2 hours in buffer containing 2 mM ATP, 2 mM MgCl_2_, 1 mM EDTA, 5% glycerol, 0.01% Brij-35, 0.1% 2-mercaptoethanol, 0.25 mM DTT, and 10 mM MOPS/NaOH (pH 7.0). Aliquots of the reaction mixture were subjected to SDS-PAGE, transferred to nitrocellulose, stained with fast green, and blotted with anti-phospho-S70-BCL2 or subjected to trypsin digestion followed by nano-LC/MS-MS using an Thermo Ultimate 3000 RSLCnano HPLC system coupled with a Thermo Scientific QExactive HF-X mass spectrometer (Thermo Scientific, Bremen, Germany) to identify phosphorylation sites (Mayo Clinic Proteomics Core, Rochester, MN).

### Surface plasmon resonance (SPR)

All proteins for SPR were concentrated in a centrifugal concentrator (Centricon; EMD Millipore), dialyzed against Biacore buffer [150 mM NaCl, 0.05 mM EDTA, 0.005% [wt/vol] Polysorbate 20, and 10 mM Hepes (pH 7.4)], and stored at 4°C for <48 h before use. Binding assays were performed at 25°C on a Biacore T200 SPR analyzer (GE Healthcare). His_6_-tagged Casp8p41 protein (0.2 mg/mL) was immobilized on CM5 S chip (BR-1000-34) at a flow of 10 μL/min and reached a level of 6000–7000 resonance units (RU). BCL2 or phospho-S70-BCL2 at concentrations from 0–500 nM in Biacore buffer was passed over the chip surface at flow rate of 30 μl/min for 90 s, allowed to dissociate for 10 min and then desorbed with consecutive injections of 2 M MgCl_2_ and 1 M NaCl. Kinetic analysis of SPR data was performed using BiaEvaluation (GE). Sensograms were subtracted for background contributions, and affinity constants were derived using a steady state affinity fitting 1:1 interaction model.

### Western blot analysis

Prior to treatment, cells cultured for use in the phospho-protein blots were cultured overnight in RPMI-AB. Cell lysates were run on 10% or 12% SDS-PAGE and transferred to PVDF membrane. Blots for phospho-proteins were blocked with 5% BSA in Tris-buffered saline with 0.2% Tween (TBST) or blocked with 5% dried milk in TBST. Following blocking, primary antibody was added to blocking solution and blots incubated for at least two hours. The membranes were then washed and probed with a secondary antibody conjugated to HRP in blocking solution for at least an hour, washed again and exposed using SuperSignal West Pico PLUS chemiluminescent substrate. When re-probed, membranes were first stripped using 6 M guananine HCl [65].

The following antibodies were used for probing: Phospho-S70-BCL2 [Clone 5H2] rabbit mAb, phospho-ERK1/2 (P-p44/42 MAPK) (T202/Y204) [clone D13.14.4E XP(R)] rabbit mAb, ERK1/2 (p44/42 MAPK) [Clone L34F12] mouse mAb, phospho-SAPK/JNK (T183/Y185) [Clone G9] mouse mAb, SAPK/JNK rabbit Ab, and GAPDH [Clone 14C10] rabbit mAb from Cell Signaling Technology (Danvers, MA); and STAT1 [Clone E-23X] rabbit Ab and BCL2 [C21] rabbit polyclonal IgG from Santa Cruz Biotechnology (Dallas, TX).

### Immunoprecipitation

Activated CD4 cells (as mentioned above) were transfected with Casp8p41-HA expression vector using Lanza Nucleofector IIb system. One hour after electroporation, cells were treated with Z-VAD-fmk (10 μM) and Ixazomib (100 nM) for 4 h (to prevent protein degradation and cell death induced by Casp8p41 expression), followed by treatment with or without bryostatin (10 ng/ml) for an additional 1 h. Cells were collected and washed and lysis with cell lysis buffer (20 mM Tris, pH 7.5, 150 mM NaCl, 1.0% Tween-20, protease and phosphatases inhibitors). Five hundred micrograms of total cell lysate were diluted in 500 μl of buffer, precleared with 25 μl Protein A/G agarose (Santa Cruz). Precleared lysate incubated with Anti-HA matrix (cat # 11815016001, Roche, Germany) or 2 μg anti–BCL2 (SC-509, Santa Cruz) or mouse IgG for overnight at 4C. After incubation, 20 μl of protein A/G agarose was added to the appropriated samples, and complexes were washed five times in IP buffer (20 mM Tris, pH 7.5, 300 mM NaCl, 1.0% Tween-20, protease and phosphatases inhibitors) and boiled in 20 μl 2× SDS-PAGE loading buffer, subjected to SDS-PAGE, and immunoblotted with HA-HRP antibody (Roche) or BCL2 antibody.

### Flow cytometry

For experiments measuring BCL2 in cellular subsets, PBMCs were washed in PBS and then stained for surface markers. Following surface staining, cells were fixed in 2% paraformaldehyde, washed in PBS, resuspended in a staining buffer (0.1% NP40, 5% BSA in PBS) and stained for intracellular markers (BCL2). The following reagents were used for staining: Live/Dead Fixable Aqua Dead Cell Stain Kit [Invitrogen, Waltham, MA], mouse anti-human CD3-AF700 [BD Biosciences, San Diego, CA/Clone SP34-2), mouse anti-human CD4-APC [eBioscience, Watham, MA/Clone RPA-T4], mouse anti-human CD8-Pacific Blue [BD Bioscience/Clone RPA-T8], mouse anti-human CD27-PE [BD Biosciences/Clone M-T271], mouse anti-human CD45RO-ECD [Beckman Coulter, Brea, CA/Clone UCHL1], mouse anti-human BCL2 FITC [Dako/Clone 124], or normal mouse IgG1 FITC [Santa Cruz Biotechnology, Inc.]. Following staining for 1 hour, cells were washed in PBS and fixed in 2% PFA.

For experiments examining p24 and BCL2 simultaneously, PBMCs from HIV positive subjects were fixed with 2% PFA, washed with PBS, and permeabilized with staining buffer. The following antibodies were then used for staining in addition to the afore-mentioned BCL2 antibody and mouse IgG_1_ FITC: Normal Mouse IgG_1_ PE [Santa Cruz Biotechnology, Inc.] and mouse p24-PE [Beckman Coulter/KC57-RD1]. Following staining for 1 hour, cells were washed in PBS and fixed in 2% PFA. 20,000 events per sample were collected.

Flow cytometry was performed using BD FortessaX20, BD LSRII or BD FACSCalibur flow cytometers, and results were analyzed with FlowJo v10.2 software (Tree Star, Inc).

### Casp8p41 transfection

Primary CD4+ T cells from healthy donors were treated with LRA for 20 hours and transfected with plasmids encoding empty vector-GFP or Casp8p41-GFP [18] using an Amaxa Human T Cell Nucleofector kit (Lonza Bioscience, Basel, Switzerland) per the manufacturer’s protocol. Transfected cells were transferred back into the conditioned medium.

### Single-Cell RNA-Seq data processing and integration analyses

cDNA libraries (prepared with 10X Genomics single cell reagent 3’ v2) were sequenced on an Illumina HiSeq 4000 with paired-end 100bp reads. 10X Genomics Cell Ranger Single Cell Software Suite (v2.2.0) was used to demultiplex raw base call (BCL) files generated from the sequencer into FASTQ files and to perform alignment to the GRCh38 genome, filtering, barcode counting and UMI counting. This analysis was done at the sample level and the output files were used for downstream analyses.

Integrated analysis of two samples was performed in R using the Seurat package (v2.0) [66]. Genes expressed in fewer than 3 cells and cells with <200 or >3500 genes and more than 40% mitochondrial genes were excluded for subsequent analysis in each sample. Each dataset was normalized with a scale factor of 10^4^ and log_2_-transformed. Common sources of variation between the two datasets were identified in a shared correlation space by performing a Canonical Correlation Analysis (CCA) on the common highly variable genes (HVGs) of the two preprocessed dataset (top 1000 in each dataset). We used the function FindClusters to implement a graph-based clustering algorithm on the first 20 Canonical Correlation vectors (CCs) with resolution of 0.4, 0.6 and 0.8 and a resolution of 0.6 was chosen as optimal for the analysis. t-distributed stochastic neighbor embedding (t-SNE) plots were used to visualize a two-dimensional representation of the cell types. We used the function FindConservedMarkers to identify enriched genes in each cluster that were conserved across the two conditions and the function FindMarkers to find genes in a cluster that are differentially expressed between the two samples.

### Statistical analysis

Graphs were made using GraphPad Prism 6 (GraphPad, Inc) and present means ± the standard deviation unless otherwise stated. tSNE plots were made using Loupe Cell Browser 2.0 (10X Genomics, Inc.). Statistical analyses were performed using GraphPad Prism and the types of test used for analysis are listed in the corresponding figure legends. P values < 0.05 were considered significant. Gene ontology was performed using the PANTHER overrepresentation test and Fischer’s Exact test and the Bonferroni correction for multiple testing.

## References

[1] Deeks SG. HIV: Shock and kill. Nature. 2012;487(7408):439–40. 10.1038/487439a 22836995

[2] Zerbato JM, Purves HV, Lewin SR, Rasmussen TA. Between a shock and a hard place: challenges and developments in HIV latency reversal. Curr Opin Virol. 2019;38:1–9. 10.1016/j.coviro.2019.03.004 31048093 PMC6819240

[3] Archin NM, Kirchherr JL, Sung JA, Clutton G, Sholtis K, Xu Y, et al Interval dosing with the HDAC inhibitor vorinostat effectively reverses HIV latency. J Clin Invest. 2017;127(8):3126–35. 10.1172/JCI92684 28714868 PMC5531421

[4] Elliott JH, Wightman F, Solomon A, Ghneim K, Ahlers J, Cameron MJ, et al Activation of HIV Transcription with Short-Course Vorinostat in HIV-Infected Patients on Suppressive Antiretroviral Therapy. PLoS Pathog. 2014;10(10):e1004473 10.1371/journal.ppat.1004473 25393648 PMC4231123

[5] Gutierrez C, Serrano-Villar S, Madrid-Elena N, Perez-Elias MJ, Martin ME, Barbas C, et al Bryostatin-1 for latent virus reactivation in HIV-infected patients on antiretroviral therapy. Aids. 2016;30(9):1385–92. 10.1097/QAD.0000000000001064 26891037

[6] Rasmussen TA, Tolstrup M, Brinkmann CR, Olesen R, Erikstrup C, Solomon A, et al Panobinostat, a histone deacetylase inhibitor, for latent-virus reactivation in HIV-infected patients on suppressive antiretroviral therapy: a phase 1/2, single group, clinical trial. Lancet HIV. 2014;1(1):e13–21. 10.1016/S2352-3018(14)70014-1 26423811

[7] Sogaard OS, Graversen ME, Leth S, Olesen R, Brinkmann CR, Nissen SK, et al The Depsipeptide Romidepsin Reverses HIV-1 Latency In Vivo. PLoS Pathog. 2015;11(9):e1005142 10.1371/journal.ppat.1005142 26379282 PMC4575032

[8] Doitsh G, Galloway NL, Geng X, Yang Z, Monroe KM, Zepeda O, et al Cell death by pyroptosis drives CD4 T-cell depletion in HIV-1 infection. Nature. 2014;505(7484):509–14. 10.1038/nature12940 24356306 PMC4047036

[9] Cummins NW, Badley AD. Mechanisms of HIV-associated lymphocyte apoptosis: 2010. Cell Death Dis. 2010;1:e99 10.1038/cddis.2010.77 21368875 PMC3032328

[10] Cooper A, Garcia M, Petrovas C, Yamamoto T, Koup RA, Nabel GJ. HIV-1 causes CD4 cell death through DNA-dependent protein kinase during viral integration. Nature. 2013;498(7454):376–9. 10.1038/nature12274 23739328

[11] Cummins NW, Badley AD. Casp8p41 and HIV. Oncotarget. 2015;6(27):23042–3. 10.18632/oncotarget.5238 26309081 PMC4695101

[12] Strack PR, Frey MW, Rizzo CJ, Cordova B, George HJ, Meade R, et al Apoptosis mediated by HIV protease is preceded by cleavage of Bcl-2. Proc Natl Acad Sci U S A. 1996;93(18):9571–6. 10.1073/pnas.93.18.9571 8790371 PMC38469

[13] Nie Z, Phenix BN, Lum JJ, Alam A, Lynch DH, Beckett B, et al HIV-1 protease processes procaspase 8 to cause mitochondrial release of cytochrome c, caspase cleavage and nuclear fragmentation. Cell Death Differ. 2002;9(11):1172–84. 10.1038/sj.cdd.4401094 12404116

[14] Nie Z, Bren GD, Vlahakis SR, Schimnich AA, Brenchley JM, Trushin SA, et al Human immunodeficiency virus type 1 protease cleaves procaspase 8 in vivo. J Virol. 2007;81(13):6947–56. 10.1128/JVI.02798-06 17442709 PMC1933285

[15] Nie Z, Bren GD, Rizza SA, Badley AD. HIV Protease Cleavage of Procaspase 8 is Necessary for Death of HIV-Infected Cells. Open Virol J. 2008;2:1–7. 10.2174/1874357900802010001 18818774 PMC2548307

[16] Sainski AM, Natesampillai S, Cummins NW, Bren GD, Taylor J, Saenz DT, et al The HIV-1-specific protein Casp8p41 induces death of infected cells through Bax/Bak. J Virol. 2011;85(16):7965–75. 10.1128/JVI.02515-10 21653671 PMC3147983

[17] Sainski AM, Dai H, Natesampillai S, Pang YP, Bren GD, Cummins NW, et al Casp8p41 generated by HIV protease kills CD4 T cells through direct Bak activation. J Cell Biol. 2014;206(7):867–76. 10.1083/jcb.201405051 25246614 PMC4178959

[18] Cummins NW, Sainski AM, Dai H, Natesampillai S, Pang YP, Bren GD, et al Prime, Shock, and Kill: Priming CD4 T Cells from HIV Patients with a BCL-2 Antagonist before HIV Reactivation Reduces HIV Reservoir Size. J Virol. 2016;90(8):4032–48. 10.1128/JVI.03179-15 26842479 PMC4810548

[19] Davenport MP, Khoury DS, Cromer D, Lewin SR, Kelleher AD, Kent SJ. Functional cure of HIV: the scale of the challenge. Nat Rev Immunol. 2019;19(1):45–54. 10.1038/s41577-018-0085-4 30410126

[20] Mikulak J, Oriolo F, Zaghi E, Di Vito C, Mavilio D. Natural killer cells in HIV-1 infection and therapy. Aids. 2017;31(17):2317–30. 10.1097/QAD.0000000000001645 28926399 PMC5892189

[21] Jiang G, Dandekar S. Targeting NF-kappaB signaling with protein kinase C agonists as an emerging strategy for combating HIV latency. AIDS Res Hum Retroviruses. 2015;31(1):4–12. 10.1089/AID.2014.0199 25287643 PMC4287114

[22] Natesampillai S, Cummins NW, Nie Z, Sampath R, Baker JV, Henry K, et al HIV Protease-Generated Casp8p41, When Bound and Inactivated by Bcl2, Is Degraded by the Proteasome. J Virol. 2018;92(13). 10.1128/JVI.00037-18 PMC6002723 29643240

[23] Zhao M, De Crignis E, Rokx C, Verbon A, van Gelder T, Mahmoudi T, et al T cell toxicity of HIV latency reversing agents. Pharmacol Res. 2019;139:524–34. 10.1016/j.phrs.2018.10.023 30366100

[24] Hiscott J, Kwon H, Genin P. Hostile takeovers: viral appropriation of the NF-kappaB pathway. J Clin Invest. 2001;107(2):143–51. 10.1172/JCI11918 11160127 PMC199181

[25] Kucharczak J, Simmons MJ, Fan Y, Gelinas C. To be, or not to be: NF-kappaB is the answer—role of Rel/NF-kappaB in the regulation of apoptosis. Oncogene. 2003;22(56):8961–82. 10.1038/sj.onc.1207230 14663476

[26] Jordan A, Bisgrove D, Verdin E. HIV reproducibly establishes a latent infection after acute infection of T cells in vitro. Embo J. 2003;22(8):1868–77. 10.1093/emboj/cdg188 12682019 PMC154479

[27] Spina CA, Anderson J, Archin NM, Bosque A, Chan J, Famiglietti M, et al An in-depth comparison of latent HIV-1 reactivation in multiple cell model systems and resting CD4+ T cells from aviremic patients. PLoS Pathog. 2013;9(12):e1003834 10.1371/journal.ppat.1003834 24385908 PMC3873446

[28] Eischen CM, Kottke TJ, Martins LM, Basi GS, Tung JS, Earnshaw WC, et al Comparison of Apoptosis in Wild-type and Fas-resistant Cells: Chemotherapy-induced Apoptosis Is Not Dependent on Fas/Fas Ligand Interactions. Blood. 1997;90(3):935–43. 9242521

[29] Newton K, Strasser A. Ionizing Radiation and Chemotherapeutic Drugs Induce Apoptosis in Lymphocytes in the Absence of Fas or FADD/MORT1 Signaling. Implications for Cancer Therapy. Journal of Experimental Medicine. 2000;191(1):195–200. 10.1084/jem.191.1.195 10620618 PMC2195793

[30] Earnshaw WC, Martins LM, Kaufmann SH. Mammalian Caspases: Structure, Activation, Substrates and Functions During Apoptosis. Annual Review of Biochemistry. 1999;68:383–424. 10.1146/annurev.biochem.68.1.383 10872455

[31] Slee EA, Adrain C, Martin SJ. Serial Killers: Ordering Caspase Activation Events in Apoptosis. Cell Death and Differentiation. 1999;6(11):1067–74. 10.1038/sj.cdd.4400601 10578175

[32] Katsikis PD, Wunderlich ES, Smith CA, Herzenberg LA, Herzenberg LA. Fas antigen stimulation induces marked apoptosis of T lymphocytes in human immunodeficiency virus-infected individuals. J Exp Med. 1995;181(6):2029–36. 10.1084/jem.181.6.2029 7539037 PMC2192074

[33] Westendorp MO, Frank R, Ochsenbauer C, Stricker K, Dhein J, Walczak H, et al Sensitization of T cells to CD95-mediated apoptosis by HIV-1 Tat and gp120. Nature. 1995;375(6531):497–500. 10.1038/375497a0 7539892

[34] Dai H, Ding H, Meng XW, Lee SH, Schneider PA, Kaufmann SH. Contribution of Bcl-2 phosphorylation to Bak binding and drug resistance. Cancer Res. 2013;73(23):6998–7008. 10.1158/0008-5472.CAN-13-0940 24097825 PMC3910374

[35] Ruvolo PP, Deng X, May WS. Phosphorylation of Bcl2 and regulation of apoptosis. Leukemia. 2001;15(4):515–22. 10.1038/sj.leu.2402090 11368354

[36] May WS, Tyler PG, Ito T, Armstrong DK, Qatsha KA, Davidson NE. Interleukin-3 and bryostatin-1 mediate hyperphosphorylation of BCL2 alpha in association with suppression of apoptosis. J Biol Chem. 1994;269(43):26865–70. 7929424

[37] Ruvolo PP, Deng X, Carr BK, May WS. A functional role for mitochondrial protein kinase Calpha in Bcl2 phosphorylation and suppression of apoptosis. J Biol Chem. 1998;273(39):25436–42. 10.1074/jbc.273.39.25436 9738012

[38] Deng X, Ruvolo P, Carr B, May WS Jr., Survival function of ERK1/2 as IL-3-activated, staurosporine-resistant Bcl2 kinases. Proc Natl Acad Sci U S A. 2000;97(4):1578–83. 10.1073/pnas.97.4.1578 10677502 PMC26477

[39] Evenou JP, Wagner J, Zenke G, Brinkmann V, Wagner K, Kovarik J, et al The potent protein kinase C-selective inhibitor AEB071 (sotrastaurin) represents a new class of immunosuppressive agents affecting early T-cell activation. J Pharmacol Exp Ther. 2009;330(3):792–801. 10.1124/jpet.109.153205 19491325

[40] Toullec D, Pianetti P, Coste H, Bellevergue P, Grand-Perret T, Ajakane M, et al The bisindolylmaleimide GF 109203X is a potent and selective inhibitor of protein kinase C. J Biol Chem. 1991;266(24):15771–81. 1874734

[41] Morris EJ, Jha S, Restaino CR, Dayananth P, Zhu H, Cooper A, et al Discovery of a novel ERK inhibitor with activity in models of acquired resistance to BRAF and MEK inhibitors. Cancer Discov. 2013;3(7):742–50. 10.1158/2159-8290.CD-13-0070 23614898

[42] Bennett BL, Sasaki DT, Murray BW, O'Leary EC, Sakata ST, Xu W, et al SP600125, an anthrapyrazolone inhibitor of Jun N-terminal kinase. Proc Natl Acad Sci U S A. 2001;98(24):13681–6. 10.1073/pnas.251194298 11717429 PMC61101

[43] Kim Y, Anderson JL, Lewin SR. Getting the "Kill" into "Shock and Kill": Strategies to Eliminate Latent HIV. Cell Host Microbe. 2018;23(1):14–26. 10.1016/j.chom.2017.12.004 29324227 PMC5990418

[44] Li Q, Cheng L, Shen K, Jin H, Li H, Cheng Y, et al Efficacy and Safety of Bcl-2 Inhibitor Venetoclax in Hematological Malignancy: A Systematic Review and Meta-Analysis of Clinical Trials. Front Pharmacol. 2019;10:697 10.3389/fphar.2019.00697 31293422 PMC6598635

[45] Pal D, Basu A. Protein kinase C-eta regulates Mcl-1 level via ERK1. Cell Signal. 2017;40:166–71. 10.1016/j.cellsig.2017.09.012 28939105

[46] Amigo-Jimenez I, Bailon E, Aguilera-Montilla N, Terol MJ, Garcia-Marco JA, Garcia-Pardo A. Bone marrow stroma-induced resistance of chronic lymphocytic leukemia cells to arsenic trioxide involves Mcl-1 upregulation and is overcome by inhibiting the PI3Kdelta or PKCbeta signaling pathways. Oncotarget. 2015;6(42):44832–48. 10.18632/oncotarget.6265 26540567 PMC4792595

[47] Saraiva L, Silva RD, Pereira G, Goncalves J, Corte-Real M. Specific modulation of apoptosis and Bcl-xL phosphorylation in yeast by distinct mammalian protein kinase C isoforms. J Cell Sci. 2006;119(Pt 15):3171–81. 10.1242/jcs.03033 16835272

[48] Manicassamy S, Gupta S, Huang Z, Sun Z. Protein kinase C-theta-mediated signals enhance CD4+ T cell survival by up-regulating Bcl-xL. J Immunol. 2006;176(11):6709–16. 10.4049/jimmunol.176.11.6709 16709830

[49] Archin NM, Liberty AL, Kashuba AD, Choudhary SK, Kuruc JD, Crooks AM, et al Administration of vorinostat disrupts HIV-1 latency in patients on antiretroviral therapy. Nature. 2012;487(7408):482–5. 10.1038/nature11286 22837004 PMC3704185

[50] Zheng L, Yang Y, Guocai L, Pauza CD, Salvato MS. HIV Tat protein increases Bcl-2 expression in monocytes which inhibits monocyte apoptosis induced by tumor necrosis factor-alpha-related apoptosis-induced ligand. Intervirology. 2007;50(3):224–8. 10.1159/000100565 17356300 PMC2384232

[51] Mbita Z, Hull R, Dlamini Z. Human immunodeficiency virus-1 (HIV-1)-mediated apoptosis: new therapeutic targets. Viruses. 2014;6(8):3181–227. 10.3390/v6083181 25196285 PMC4147692

[52] Regamey N, Harr T, Battegay M, Erb P. Downregulation of Bcl-2, but not of Bax or Bcl-x, is associated with T lymphocyte apoptosis in HIV infection and restored by antiretroviral therapy or by interleukin 2. AIDS Res Hum Retroviruses. 1999;15(9):803–10. 10.1089/088922299310700 10381168

[53] Airo P, Torti C, Uccelli MC, Malacarne F, Palvarini L, Carosi G, et al Naive CD4+ T lymphocytes express high levels of Bcl-2 after highly active antiretroviral therapy for HIV infection. AIDS Res Hum Retroviruses. 2000;16(17):1805–7. 10.1089/08892220050195766 11118066

[54] Ehrhard S, Wernli M, Kaufmann G, Pantaleo G, Rizzardi GP, Gudat F, et al Effect of antiretroviral therapy on apoptosis markers and morphology in peripheral lymph nodes of HIV-infected individuals. Infection. 2008;36(2):120–9. 10.1007/s15010-008-7368-9 18379725

[55] Grelli S, Balestrieri E, Matteucci C, Minutolo A, D'Ettorre G, Lauria F, et al Apoptotic cell signaling in lymphocytes from HIV+ patients during successful therapy. Ann N Y Acad Sci. 2006;1090:130–7. 10.1196/annals.1378.014 17384255

[56] Cummins NW, Badley AD. Anti-apoptotic mechanisms of HIV: lessons and novel approaches to curing HIV. Cellular and molecular life sciences: CMLS. 2013;70(18):3355–63. 10.1007/s00018-012-1239-3 23275944 PMC3753464

[57] Mehla R, Bivalkar-Mehla S, Zhang R, Handy I, Albrecht H, Giri S, et al Bryostatin modulates latent HIV-1 infection via PKC and AMPK signaling but inhibits acute infection in a receptor independent manner. PLoS One. 2010;5(6):e11160 10.1371/journal.pone.0011160 20585398 PMC2886842

[58] Perez M, de Vinuesa AG, Sanchez-Duffhues G, Marquez N, Bellido ML, Munoz-Fernandez MA, et al Bryostatin-1 synergizes with histone deacetylase inhibitors to reactivate HIV-1 from latency. Curr HIV Res. 2010;8(6):418–29. 10.2174/157016210793499312 20636281

[59] Dietz AB, Bulur PA, Emery RL, Winters JL, Epps DE, Zubair AC, et al A novel source of viable peripheral blood mononuclear cells from leukoreduction system chambers. Transfusion. 2006;46(12):2083–9. 10.1111/j.1537-2995.2006.01033.x 17176319

[60] Crawford TQ, Jalbert E, Ndhlovu LC, Barbour JD. Concomitant evaluation of PMA+ionomycin-induced kinase phosphorylation and cytokine production in T cell subsets by flow cytometry. Cytometry A. 2014;85(3):268–76. 10.1002/cyto.a.22444 24464647 PMC13472587

[61] Postler TS, Pantry SN, Desrosiers RC, Ghosh S. Identification and characterization of a long non-coding RNA up-regulated during HIV-1 infection. Virology. 2017;511:30–9. 10.1016/j.virol.2017.08.006 28803142 PMC5623643

[62] Ramalingam SS, Kummar S, Sarantopoulos J, Shibata S, LoRusso P, Yerk M, et al Phase I study of vorinostat in patients with advanced solid tumors and hepatic dysfunction: a National Cancer Institute Organ Dysfunction Working Group study. J Clin Oncol. 2010;28(29):4507–12. 10.1200/JCO.2010.30.2307 20837947 PMC2988640

[63] Zerbato JM, McMahon DK, Sobolewski MD, Mellors JW, Sluis-Cremer N. Naive CD4+ T Cells Harbor a Large Inducible Reservoir of Latent, Replication-Competent HIV-1. Clin Infect Dis. 2019. 10.1093/cid/ciz108 PMC6853701 30753360

[64] Dai H, Meng XW, Lee SH, Schneider PA, Kaufmann SH. Context-dependent Bcl-2/Bak interactions regulate lymphoid cell apoptosis. J Biol Chem. 2009;284(27):18311–22. 10.1074/jbc.M109.004770 19351886 PMC2709361

[65] Kaufmann SH, Ewing CM, Shaper JH. The erasable Western blot. Anal Biochem. 1987;161(1):89–95. 10.1016/0003-2697(87)90656-7 3578791

[66] Butler A, Hoffman P, Smibert P, Papalexi E, Satija R. Integrating single-cell transcriptomic data across different conditions, technologies, and species. Nat Biotechnol. 2018;36(5):411–20. 10.1038/nbt.4096 29608179 PMC6700744

